# Academic Nurse-Managed Community Clinics Transitioning to Telehealth: Case Report on the Rapid Response to COVID-19

**DOI:** 10.2196/24521

**Published:** 2020-12-01

**Authors:** Rebecca Sutter, Alison E Cuellar, Megan Harvey, Y Alicia Hong

**Affiliations:** 1 School of Nursing College of Health and Human Services George Mason University Fairfax, VA United States; 2 Department of Health Administration and Policy College of Health and Human Services George Mason University Fairfax, VA United States

**Keywords:** telehealth, telemedicine, COVID-19, nurse practitioners, safety net clinics, community clinics, nurse, clinic, transition

## Abstract

**Background:**

In response to the COVID-19 pandemic, many health care organizations have adopted telehealth. The current literature on transitioning to telehealth has mostly been from large health care or specialty care organizations, with limited data from safety net or community clinics.

**Objective:**

This is a case report on the rapid implementation of a telehealth hub at an academic nurse-managed community clinic in response to the national COVID-19 emergency. We also identify factors of success and challenges associated with the transition to telehealth.

**Methods:**

This study was conducted at the George Mason University Mason and Partners clinic, which serves the dual mission of caring for community clinic patients and providing health professional education. We interviewed the leadership team of Mason and Partners clinics and summarized our findings.

**Results:**

Mason and Partners clinics reacted quickly to the COVID-19 crisis and transitioned to telehealth within 2 weeks of the statewide lockdown. Protocols were developed for a coordination hub, a main patient triage and appointment telephone line, a step-by-step flowchart of clinical procedure, and a team structure with clearly defined work roles and backups. The clinics were able to maintain most of its clinical service and health education functions while adapting to new clinic duties that arose during the pandemic.

**Conclusions:**

The experiences learned from the Mason and Partners clinics are transferable to other safety net clinics and academic nurse-led community clinics. The changes arising from the pandemic have resulted in sustainable procedures, and these changes will have a long-term impact on health care delivery and training.

## Introduction

The COVID-19 pandemic has been rapidly transforming the US health care system. Within a few weeks of the lockdown, telehealth has been deployed across the country at a breathtaking speed. Prior to the pandemic, telehealth growth was incremental, and as of 2019, telehealth services have only been used by 8% of Americans [1]. Under the COVID-19 crisis, US insurers have quickly expanded coverage to include all telehealth visit types, including those from home [2]. Most states have relaxed their licensure requirements for care delivered across state boundaries [3]. In addition to allowing for the broad reimbursement of televisits, the Department of Health and Human Services has waived the enforcement of Health Insurance Portability and Accountability Act (HIPAA) regulations to allow the use of consumer audio and video communication for telehealth visits [3].

With these changes, telehealth is being leveraged with enormous speed and scale, turning into the forward front line in the battle against the pandemic [4]. In the emerging literature on telehealth, most studies have been focused on telemental health [5] or highly specialized health care, such as neuropathology and palliative care [6,7], and these studies typically come from large health care organizations [8]. To date, almost no studies have reported on the adoption of telehealth in academic nurse-managed community clinics, which serve the dual mission of training health professionals and caring for communities.

Prior to the pandemic, the adoption of telehealth in academic nurse-managed community clinics was slow for 2 reasons. First, health professional training requires substantial interaction time between faculty members and students. The procedures for facilitating teaching during clinical encounters have not been developed to encompass the audio-visual interface of telehealth. Second, these community clinics typically serve vulnerable populations, such as immigrants and low-income residents without health insurance. Many of these patients are slow adopters of eHealth tools, and some have limited access to high-speed internet [9,10]. This study aimed to address the current literature gap with the following objectives: (1) document the rapid implementation of a telehealth hub in an academic nurse-managed community clinic in response to the national COVID-19 emergency, (2) identify challenges and key factors for the successful transition to and dissemination of telehealth in these community clinics, and (3) explore strategies that ensure the sustainability of the telehealth program in these community clinics and their dual mission of providing treatment and teaching health professionals.

## Methods

### Study Setting

The academic nurse-managed clinic described in this study is the George Mason University Mason and Partners clinic. Since their establishment in 2013, Mason and Partners clinics have served the uninsured and immigrant communities within the Prince William and Fairfax counties in Northern Virginia. Across 10 sites, these clinics offer a bridge-care model, providing health care, school physicals, screenings, immunizations, and mental health services for vulnerable populations located in low-income and medically underserved areas. Nurse practitioners who have full independent practice authority in Virginia provide care in teams comprised of registered nurses, social workers, and students at undergraduate, graduate, and doctoral levels. The teams provide direct care, prescription and medication management, chronic care management through patient panels, and referrals to community services. Prior to the pandemic, Mason and Partners clinics served over 4000 patients annually and trained more than 250 nursing, social work, public health, health administration, and health informatics students a year. In 2019, prior to the pandemic, Mason and Partners clinics received federal funding for telehealth equipment with a focus on chronic disease and substance use disorder.

### Data Collection and Analysis

We conducted semistructured individual interviews with the key operational and clinical staff of Mason and Partners clinics involved in the telehealth transition. An interview guide was prepared priori, covering topics on the transition process, challenges and solutions during the transition, lessons learned, and next steps. All interviews were conducted via web-based meeting interface and lasted for 30-60 minutes. Participants gave oral consent before the interview. During the interviews, we took detailed notes, which were summarized into major themes. The study protocol was approved by the Institutional Review Board of George Mason University.

## Results

### Identification of Needs and Decision Making

On March 16, 2020, George Mason University suspended all on-campus activities and transitioned to web-based education. Mason and Partners clinics suspended clinical operations in local sites, such as public schools and county specialty behavioral health centers, which had been shut down. Clinic buildings were also closed. The clinic leadership team held emergency conference calls to discuss a contingency plan. The Mason and Partners team recognized the following needs: (1) continuous health care would be critical for Mason and Partners patients, given their lack of insurance and alternative sources of care, high prevalence of chronic conditions, and social needs; (2) interprofessional health education should be continued for all health professional students; and (3) the health and safety of Mason and Partners staff was paramount, but the supply of personal protection equipment was limited. Given the pressing needs and constraints, the leadership team made the decision to transition clinic operations to telehealth operations integrated with web-based education. Mason and Partners clinics were reorganized, with 1 location continuing to accept walk-in appointments for new and current patients and the remaining clinic sites transitioning to telehealth.

### Protocol Development

After the decision to transition to telehealth was made in response to the pandemic, the leadership team developed the following clinical operation protocol: (1) the walk-in clinic was open twice a week; (2) the screening and waiting area was moved to the parking lot; (3) a staff member took patients’ temperatures and asked about their potential exposure to COVID-19; (4) patients with high temperatures and patients exposed to COVID-19 were given information on testing or referred to the emergency room; (5) patients without symptoms of COVID-19 and patients who were not exposed to COVID-19 were directed to the clinic, which they could enter from a well or sick entrance, depending on their health status; and (5) all staff members wore masks, and patients were given masks if they did not have one.

With regard to telehealth operations, the Mason and Partners leadership team used the HIPAA-compliant Zoom for Healthcare platform (Zoom Video Communications, Inc) to conduct patient consultations and team meetings. All care providers could access the electronic health record (EHR) system from home via virtual private network. Nursing students could consult with patients on Zoom under the supervision of a faculty member. It was anticipated that a telehealth visit would take longer than the average in-person visit, so the number of patients a Mason and Partners clinic could serve per day decreased.

### Current Operation of Telehealth Services

Each Mason and Partners team member has access to a tablet preloaded with HIPAA-compliant apps, consent forms, and teaching packets. Patients make appointments via a main phone line for Mason and Partners clinics. During the phone call, a staff member performs an initial assessment of the patient. Following the Centers for Disease Control and Prevention guidelines, patients with COVID-19 symptoms are directed to the emergency room for immediate care and self-quarantine. New patients are asked to go to the walk-in clinic for their first appointment, and a telehealth appointment is scheduled with current patients while providing them with instructions on how to set up Zoom for a televisit. Before the telehealth appointment, a medical assistant calls the patient, helps the patient set up Zoom, and obtains their consent for treatment. When the “room” is ready, the clinical team joins the call. During the consultation, the patient, medical assistant, and nurse practitioner student provider have their webcams on; the nurse practitioner faculty supervisor and 2-3 students are silent observers that have their webcams turned off and audio muted. Each consultation lasts 45-60 minutes, including the initial set-up period.

A typical day at the Mason and Partners clinic begins with a prehuddle, which is when the team meets on Zoom to discuss the workload and plan the day. The day ends with a posthuddle, which is when the team exchanges thoughts and reviews the cases of the day. The telehealth team consists of an administrative lead, nurse practitioner student, nurse practitioner faculty members, and 2-3 interprofessional students (ie, social work, psychology, health informatics, and health administration students). The administrative lead organizes huddles and provides administrative and technical assistance for the team. All clinic teams have umbrella support from a social work lead and subject matter experts.

### Challenges and Solutions

Several challenges were identified during the transition to telehealth at Mason and Partners clinics. First, as safety net resources shrink during the pandemic and lockdown, free community clinics are crucial for underserved communities. However, due to limited supply of personal protection equipment, only 1 Mason and Partners clinic was open for in-person appointments. Second, more than 60% of telehealth patients needed a translator, and some did not own a smart phone or computer. As such, setting up videoconferences was challenging for these patients. Bilingual medical assistants worked diligently with patients to set up video connections whenever possible and provide phone calls as an alternative. Health care providers learned to be attentive while communicating with patients via an interpreter on Zoom. Third, without direct contact with patients, nursing and other interprofessional students were learning to communicate with patients through web-based platforms. Several health services offered in the face-to-face care model, including physical assessment and point of care lab testing, are not available in telehealth. Finally, the pandemic has created the need for new service lines, including COVID-19 sample collection. In response to a local health department request, Mason and Partners clinics added a telehealth service for homeless individuals and families who are temporarily sheltered in local hotels.

### Factors for Success and Sustainability

After reflecting on the transition to telehealth, the leadership team identified factors for successful transition and sustainability. Under the current extraordinary circumstances, all Mason and Partners team members recognized the need to continue service and embraced change. Effective teams need to be adaptive, flexible, and communicative. The leadership team acted promptly in response to the crisis and crafted detailed protocols before telehealth service implementation. Early implementation underwent several Plan-Do-Act response cycles, resulting in small adjustments. Protocols were developed for a coordination hub, a main patient triage and appointment phone line, a step-by-step flowchart of clinical procedure, and a team structure with clearly defined work roles and backups. Mason and Partners strives to maintain quality service and conserve limited resources. Despite the relaxation of federal requirements, Mason and Partners clinics use HIPAA-certified telehealth equipment and a secure EHR system.

### Next Steps

At the time of manuscript writing, Mason and Partners clinics have implemented several updates. With regard to clinical practices, we have started collecting samples for free COVID-19 testing in collaboration with the state health department. A new telehealth kiosk is also being set up in a rural partner site. This kiosk is equipped with peripheral diagnostic instruments to conduct physical exams for immediate reading by the health care providers in distant locations. Once this is piloted, Mason and Partners will expand telehealth services to a larger network of rural patients and collaborative sites. Meanwhile, programs for telemental health and the social needs of underserved populations are under development. With regard to training, the interprofessional education model has been further refined to involve social work and psychology students; all health professional students will receive more training for consultations on telehealth. Mason and Partners clinics will continue the telehealth program, even after the pandemic.

## Discussion

This is one of the first case reports on an academic nurse-managed community clinic transitioning to telehealth in response to the COVID-19 pandemic. This study is limited in the following aspects: (1) since Mason and Partners clinics may not be representative of other academic nurse-managed clinics in the country, the generalizability of this study may be limited; (2) we only interviewed a small number of key informants in the transition leadership team and may have missed a few elements in the transition; and (3) due to time constraints, we were not able to assess patient outcomes, patient satisfaction, provider satisfaction, and other indicators of health care quality in the new telehealth model.

Despite these limitations, this study showcases how a safety net clinic with the dual mission of providing education and service continues to function amid shutdowns and social distancing, thereby serving vulnerable communities and easing the burden on emergency rooms. The changes initially instigated by the pandemic are likely to be sustained and become the new normal of future health care. These changes have shaped clinical practice, health professional education, telehealth technology, community outreach, and health care policy.

In the new normal of telehealth, care providers and patients are forced to communicate through the internet. For care providers, this means developing new skills in web-based rapport building, diagnosis, and counseling; it also means remotely accessing and updating EHRs and remotely operating telehealth equipment. The new health professional training curriculum needs to incorporate these new skills [11]. Telehealth workflow should also be designed to minimize team burden and improve efficiency.

The rapid implementation of telehealth relies on robust technology support. We need telehealth equipment to be secure, low cost, portable, good quality, and high utility. For example, a telehealth kiosk with diagnostic peripheral equipment enables rapid testing and diagnosis. Ideally, videoconferencing technology should integrate effective interpreting services for patients who do not speak English. Telehealth consulting interfaces should allow for easy multiparty consultation and unobtrusive observation.

As of 2018, only 34% Americans have ever accessed EHRs [12]; people with lower levels of education and income are slow in adopting eHealth tools and have been left behind in the eHealth movement [9,10,12]. As high-speed internet access has become a new social determinant of health [13], providing no-cost or subsidized internet access for low-income populations should be prioritized, as it would become a prerequisite to accessing health services [12]. In the efforts to reduce health disparities, targeted interventions are needed to improve eHealth literacy in underserved communities, especially for skills regarding the use of smart phones or tablets for telehealth.

Under the COVID-19 crisis, many telehealth-related regulations have been relaxed to allow for rapid transition. As the pandemic wanes, we will have the opportunity to revisit these regulations and weigh the flexibility needed for care providers to adapt care delivery in novel ways against the requirements for ensuring high-quality care and reduced fraud and abuse. New quality tools are also needed to look beyond face-to-face encounters, from patient experience measures to coding practices.

In conclusion, the COVID-19 pandemic has posed unique challenges to health care delivery, and telehealth is well suited for continuous health services in the social distancing era. The experience of an academic nurse-led community clinic provides an example of a safety net and educational clinic successfully institutionalizing telehealth. Despite physical distancing, Mason and Partners clinics are socially connected and continue to serve the dual mission of providing service and education.

## Abbreviations

EHR: electronic health record
HIPAA: Health Insurance Portability and Accountability Act
